# A Simple and Efficient RNA Extraction Method from Deep-Sea Hydrothermal Vent Chimney Structures

**DOI:** 10.1264/jsme2.ME17048

**Published:** 2017-11-30

**Authors:** Hisashi Muto, Yoshihiro Takaki, Miho Hirai, Sayaka Mino, Shigeki Sawayama, Ken Takai, Satoshi Nakagawa

**Affiliations:** 1 Laboratory of Marine Environmental Microbiology, Graduate School of Agriculture, Kyoto University Oiwake-cho, Kyoto 606–8502 Japan; 2 Department of Subsurface Geobiological Analysis and Research (D-SUGAR), Japan Agency for Marine-Earth Science and Technology (JAMSTEC) 2–15 Natsushima-cho, Yokosuka 237–0061 Japan; 3 Research and Development (R&D) Center for Marine Biosciences, Marine Functional Biology Group (MFbio), Japan Agency for Marine-Earth Science and Technology (JAMSTEC) 2–15 Natsushima-cho, Yokosuka 237–0061 Japan; 4 Laboratory of Microbiology, Faculty of Fisheries Sciences, Hokkaido University 3–1–1 Minato-cho, Hakodate 041–8611 Japan

**Keywords:** deep-sea hydrothermal vent, chimney structure, RNA extraction, microbial community

## Abstract

RNA-based microbiological analyses, *e.g.*, transcriptome and reverse transcription-quantitative PCR, require a relatively large amount of high quality RNA. RNA-based analyses on microbial communities in deep-sea hydrothermal environments often encounter methodological difficulties with RNA extraction due to the presence of unique minerals in and the low biomass of samples. In the present study, we assessed RNA extraction methods for deep-sea vent chimneys that had complex mineral compositions. Mineral-RNA adsorption experiments were conducted using mock chimney minerals and *Escherichia coli* total RNA solution, and showed that detectable RNA significantly decreased possibly due to adsorption onto minerals. This decrease in RNA was prevented by the addition of sodium tripolyphosphate (STPP), deoxynucleotide triphosphates (dNTPs), salmon sperm DNA, and NaOH. The addition of STPP was also effective for RNA extraction from the mixture of *E. coli* cells and mock chimney minerals when TRIzol reagent and the RNeasy column were used, but not when the RNeasy PowerSoil total RNA kit was used. A combination of STPP, TRIzol reagent, the RNeasy column, and sonication resulted in the highest RNA yield from a natural chimney. This indirect extraction procedure is simple, rapid, inexpensive, and may be used for large-scale RNA extraction.

Advances in sequencing technologies have increased the significance of culture-independent analyses in microbial ecology, in which high-throughput sequences are obtained directly from nucleic acids extracted from various microbial habitats. Many methods for nucleic acid extraction have been developed and some of them have been optimized for specific sample types and research goals (1, 16, 20). A number of DNA extraction methods for marine sediments have been evaluated in detail (1, 26). These studies provided solutions for crucial steps including the removal of PCR inhibitors, cell disruption, prevention of DNA adsorption, and release of DNA (1, 26).

Deep-sea hydrothermal fields host diverse microbial communities of high biological productivity fueled primarily by chemolithoautotrophy (11, 33). Chemolithoautotrophs utilize reductants in hydrothermal fluids and oxidants in seawater (5, 33, 46, 48). Deep-sea vent chimneys composed of complex minerals are created by the mixing of hydrothermal fluids and seawater and have steep gradients of temperature, pH, oxidation-redox potential, and various chemicals within these structures (11, 21, 28, 47). Thus, deep-sea vent chimneys prepare suitable habitats for physiologically and phylogenetically diverse chemolithotrophic microbial communities (12, 17, 23, 46, 53). A cultivation approach revealed the occurrence of diverse microbes ranging from psychrophiles to hyperthermophiles within chimney structures (31, 32, 35), and DNA-based culture-independent studies also showed the occurrence of diverse as-yet uncultivated microorganisms (42, 45, 47).

DNA-based analyses provide information on microbial communities including dead, inactive, or dormant populations (6, 14, 51), while RNA-based methods allow for more precise assessments of the composition and function of active microbial populations (3, 38). Therefore, RNA-based methods (*e.g.* a transcriptome analysis) have been applied to many microbial habitats, including soils (52), seawater, and marine sediments (9, 55). However, RNA-based methods have only been successfully applied to free-living microbial communities in deep-sea hydrothermal environments in a few studies, and this may be due to the difficulties associated with the extraction of high-quantity and -quality RNA (13, 25, 27, 44). Furthermore, RNA extraction methods for chimney habitats have not yet been evaluated. Therefore, we herein tested two RNA extraction methods and optimization protocols, and reported a simple, rapid, and cost-effective protocol for deep-sea vent chimney habitats.

## Materials and Methods

### Preparation of a mock chimney and RNA

A mock chimney was prepared by pulverizing pyrite (FeS_2_) and barite (BaSO_4_) (3:2 [w/w]) with a mortar and pestle, followed by sterilization at 230°C for 30 min. Sulfide and sulfate minerals are major constituents of deep-sea vent chimney structures (15, 24, 42). Total RNA was prepared from *Escherichia coli* cells with TRIzol reagent (Thermo Fisher Scientific, Waltham, MA) and the RNeasy mini kit (Qiagen, Hilden, Germany). When TRIzol reagent and the RNeasy column were used together, the aqueous phase after the TRIzol treatment was loaded into the RNeasy column after mixing with an equal volume of ethanol. RNA was eluted after washing processes according to the manufacturer’s instructions.

### Adsorption experiments on the mock chimney and RNA

The RNA adsorption experiment was conducted by mixing 100 μL of the RNA solution (28 ng μL^−1^), 100 μL of a potential adsorption inhibitor, and 10 mg of mock chimney powder on ice. We assessed each of the following potential inhibitors: sodium tripolyphosphate (STPP; 100 μL, 0.6 M), deoxynucleotide triphosphates (dNTPs; 100 μL, 2.5 mM each), salmon sperm DNA (100 μL, 0.8 μg μL^−1^), and NaOH (100 μL, pH 10). dNTPs, salmon sperm DNA, and NaOH were previously assessed for nucleic acid extraction from various environmental samples (26). Although STPP was not assessed by Lever *et al.* (26), we used it as a PO_4_ source because STPP is cheap and safe. One hundred microliters of diethyl pyrocarbonate (DEPC)-treated water was used as a control. After being incubated on ice for 0 h, 4 h, and 14 h, the mixture was vortexed for 2 s and centrifuged at 2,000×*g* for 20 s. The supernatant was recovered into a new tube, purified by the RNeasy column (Qiagen), and the RNA recovered was reverse transcribed into cDNA using random hexamers with the PrimeScript RT reagent kit with gDNA Eraser (TaKaRa Bio, Otsu, Japan). *E. coli* 16S rRNA was quantified by qPCR (Thermal Cycler Dice Real Time System II; TaKaRa Bio) with the primer EUB338F-U533R (2, 54) and SYBR Premix Ex Taq II (TaKaRa Bio), following the manufacturer’s instructions. PCR without reverse transcription was used as a control. A standard curve was obtained for each run using the PCR-amplified 16S rRNA gene of *E. coli*. 16S rRNA in some samples was also quantified with Bioanalyzer 2100 (Agilent Technology, Santa Clara, USA) with the RNA 6000 pico chip kit (Agilent Technology). These experiments were run in triplicate.

### RNA extraction from a mixture of the mock chimney and *E. coli* cells

*E. coli* cells were grown in LB medium and harvested by centrifugation in the late exponential growth phase. Cells (10^8^ cells) and 0.25 g of the mock chimney were mixed and then frozen at −80°C for 48 h. RNA was extracted using the RNeasy PowerSoil total RNA kit (Qiagen; formerly the RNA PowerSoil total RNA isolation kit [MO BIO Laboratories, Carlsbad, CA]) or TRIzol reagent (Thermo Fisher Scientific) and the RNeasy column (Qiagen), in the presence or absence of 100 μL of 0.6 M STPP. RNA quality (RNA integrity number, RIN) was assessed using Bioanalyzer 2100 (Agilent Technology). 16S rRNA was quantified by qPCR as described above. All experiments were run in triplicate.

### RNA extraction from a natural chimney structure

A chimney structure was obtained from the Noho site of the Sakai field (27°31.386′N, 126°59.209′E) (34), Mid-Okinawa Trough, Japan, at a depth of 1,550 m by means of the ROV Hyper-Dolphin (Dive#1860) during R/V *Natsushima* cruise NT15-13 (JAMSTEC) in August 2015. Immediately after its recovery onboard, the chimney structure was stored at −80°C until used.

The chimney sample was pulverized with a mortar and pestle to a fine powder of micron-size particles in liquid nitrogen. Total RNA was directly extracted from 1.79–1.92 g of the sample using TRIzol reagent (Thermo Fisher Scientific) and the RNeasy column (Qiagen), in the presence or absence of 100 μL of 0.6 M STPP. In addition, the mixture of the pulverized chimney structure and STPP was sonicated using TAITEC VP-050 (TAITEC, Koshigaya, Japan) at 10W for 20 s. After 30 min on ice, the supernatant was recovered, and RNA was extracted using TRIzol reagent (Thermo Fisher Scientific) and the RNeasy column (Qiagen) (indirect extraction). STPP was used in the indirect RNA extraction procedure because the supernatant potentially includes tiny particles of the chimney structure and/or RNA accidentally released from cells by sonication. RNA quality and the 16S rRNA copy number were evaluated as described above.

### 16S rRNA sequence analysis of extracted RNA

Total RNA samples from the chimney structure were reverse transcribed to cDNA as described above. The V4–V5 regions of 16S rRNA cDNA were amplified and analyzed using Illumina sequencing (MiSeq) as previously described (36). Sequences were processed using the QIIME software package (7). OTUs were selected at the 97% similarity level using UCLUST (10) and subsequently assigned to a taxon by comparisons with SILVA 119 (39) using the RDP classifier (SSU ref NR 119; http://www.arb-silva.de/no_cache/download/archive/release_119/Exports). Raw sequencing data have been deposited in GenBank/EMBL/DDBJ under accession number DRA005630.

## Results and Discussion

### RNA adsorption to the mock chimney

**Fig. 1:**
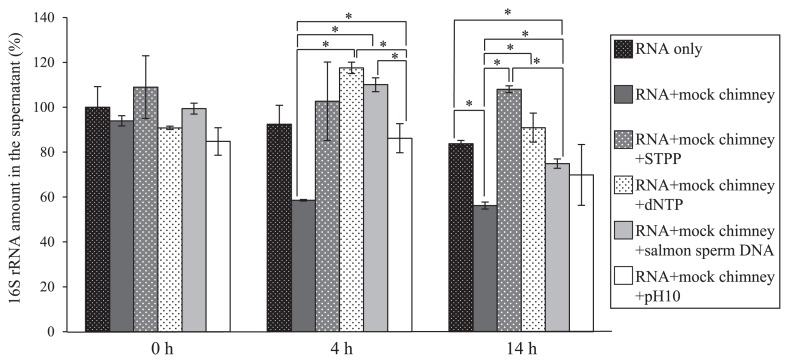
16S rRNA quantified by RT-qPCR. The 16S rRNA amount in the RNA solution without the mock chimney was defined as 100%. Values represent means from three replications±SE (*n*=3). Stars indicate significant differences between the two datasets with the *t*-test (*P*<0.05).

Mock chimney minerals were expected to trap RNA molecules to a certain extent, while some chemical treatments may prevent RNA adsorption to these minerals. We incubated mixtures of mock chimney minerals and *E. coli* total RNA solution, and then quantified the amounts of dissolved 16S rRNA in supernatants. After 4 h of mixing, dissolved RNA decreased to approximately 40% of the initial amount (Fig. 1). The RNA fragmentation pattern was examined by a Bioanalyzer electrogram and even after 14 h of mixing, no apparent fragmentation of RNA was observed (Fig. S1), suggesting that the decrease in RNA resulted from adsorption to minerals, and not from degradation. RNA adsorption to minerals was prevented by the addition of STPP, dNTPs, salmon sperm DNA, and NaOH; however, detectable RNA was significantly decreased after 14 h in the presence of salmon sperm DNA (Fig. 1). These results are consistent with previous findings showing that nucleic acid adsorption onto positively charged mineral surfaces may be prevented by phosphates, nucleic acids (4, 18, 37, 40), and alkaline pH (4, 49). Among the treatments tested in this study, we focused on STPP in subsequent experiments because it exerted some of the strongest effects, is cost-effective, and may be removed with a silica column if necessary (41).

### RNA extraction from the mock chimney

**Fig. 2:**
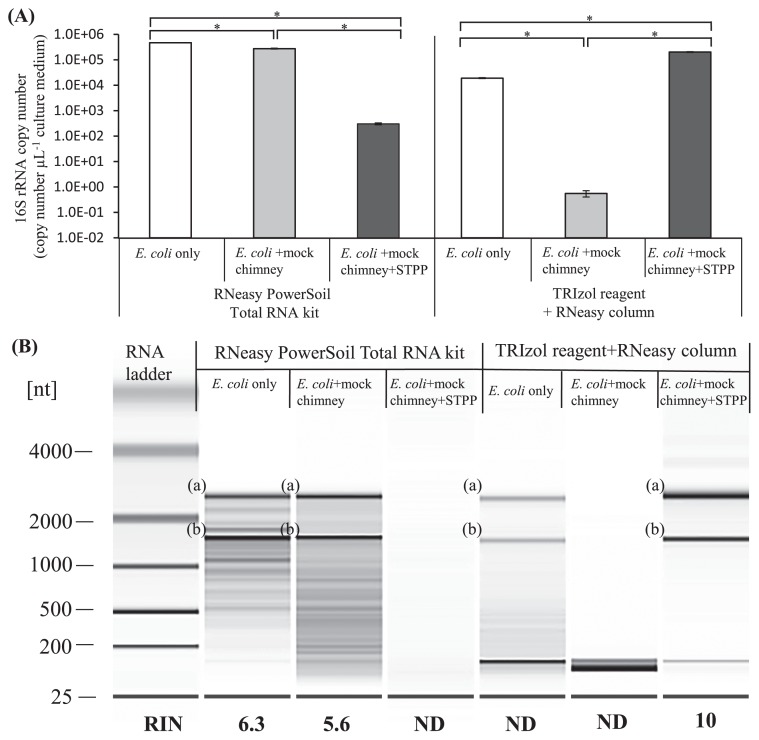
(A) RNA extraction from the mixture of the mock chimney structure and *E. coli* cells. Values represent the mean from three replications±SE (*n*=3). Stars indicate significant differences between the two data sets with the *t*-test (*P*<0.05). (B) Assessment of RNA quality using Bioanalyzer. RNA integrity number (RIN), an indicator of RNA quality, was shown. (a) and (b) indicate 23S and 16S rRNAs. ND, not determined.

The effects of STPP on RNA extraction were evaluated using a mixture of mock chimney minerals and *E. coli* cells. In the presence or absence of STPP, RNA was directly extracted by two different methods. The amount of RNA extracted significantly decreased when cells were mixed with mock chimney minerals in the absence of STPP (Fig. 2A). When TRIzol reagent and the RNeasy column were used, the amount of RNA extracted markedly decreased. In contrast, the amount of RNA extracted only slightly decreased when the RNeasy PowerSoil total RNA kit was used, even in the absence of STPP (*i.e.* the mean RNA yield [±standard error] was 4.7×10^5^ [±4.1×10^3^] copies μL^−1^ of the culture in the absence of the mock chimney and 2.8×10^5^ [±1.1×10^3^] copies μL^−1^ of the culture in the presence of the mock chimney; *n*=3) (Fig. 2A). This is potentially because RNeasy PowerSoil total RNA kit constituents may release RNA from the mineral surface; however, the chemical ingredients of the kit are not disclosed. In the presence of STPP, the amount of 16S rRNA recovered was significantly improved when TRIzol reagent and the RNeasy column were used (Fig. 2A). In contrast, when the RNeasy PowerSoil total RNA kit was used with STPP, a significantly lower amount of RNA was extracted. This is potentially because phosphate carryover interfered with the RNeasy PowerSoil total RNA kit and/or subsequent qPCR.

When combined with STPP, TRIzol reagent and the RNeasy column provided efficient RNA extraction from the mixture of mock chimney minerals and *E. coli* cells that was similar to that with the RNeasy PowerSoil total RNA kit without STPP. In addition, the quality of extracted RNA with STPP using TRIzol reagent and the RNeasy column was superior to that extracted using the RNeasy PowerSoil total RNA kit in the presence of STPP. The RNeasy PowerSoil total RNA kit resulted in RNA fragmentation and a lower RIN (43) (Fig. 2B), and this was potentially due to the bead beading step. A previous study indicated that RIN values greater than 7.0 are ideal for reproducible RT-qPCR (19). In addition, TRIzol reagent and the RNeasy column allowed RNA extraction in a shorter time (approximately 30 min) than the RNeasy PowerSoil total RNA kit (approximately 1.5 h). Although not assessed in the present study, other commercial kits, *e.g.* the NucleoSpin Soil kit (Macherey-Nagel, Düren, Germany) and Fast RNA Pro Soil-Direct kit (MP Biomedicals, Santa Ana, USA), generally take 1.5–2 h for RNA extraction. Furthermore, TRIzol reagent and the RNeasy column may reduce the cost of RNA extraction by approximately 40–80% from the commercial kits described above. TRIzol reagent and the RNeasy column may be easily used in larger scale experiments and the simultaneous extraction of RNA and DNA.

### Direct and indirect RNA extraction methods for a natural chimney sample

**Fig. 3:**
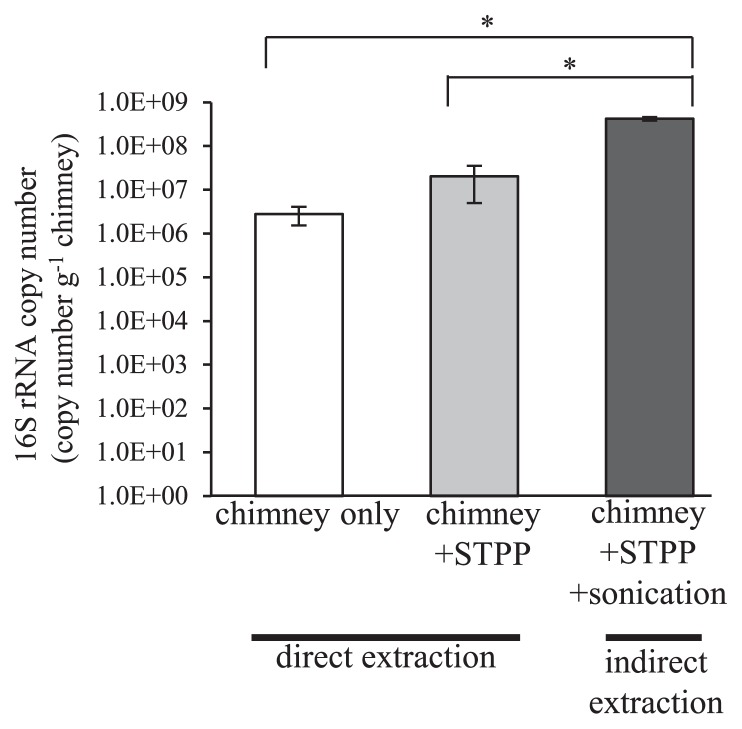
RNA extraction from an actual chimney structure with or without STPP and a sonication step. Values shown represent the mean from three replications±SE (*n*=3). Stars indicate significant differences from each other tested with the *t*-test (*P*<0.05).

A combination of TRIzol reagent and the RNeasy column was found to be a potentially effective method for RNA extraction from a natural chimney sample (Fig. 3). Although not significant, the amount of RNA extracted from the same sample was increased by the addition of STPP (Fig. 3). Since previous studies indicated the biofilm formation of microbial communities within natural chimney structures (8, 44, 48), the effects of sonication (22) during RNA extraction were also evaluated. This indirect extraction method significantly increased the amount of RNA extracted by more than 20-fold (Fig. 3), suggesting that sonication releases cells from chimney minerals and improves the efficiency of cell lysis, as previously indicated for microbial communities in sediments (29). Since the physical properties and mineral compositions of chimney structures vary in different deep-sea vents and even in different parts of the same chimney structure (50), further adjustments of the STPP amount and sonication intensity are necessary for optimization.

### Microbial 16S rRNA analysis

**Fig. 4:**
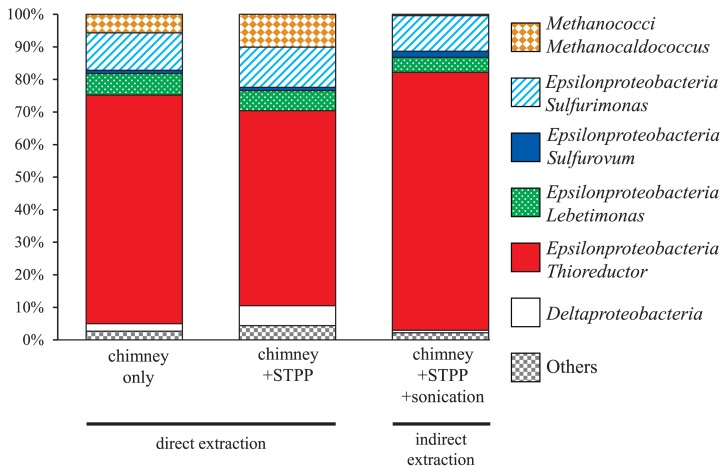
Bar graphs showing the relative abundance of 16S rRNA reads assigned to major taxonomic groups. Each color on the graph represents a distinct taxonomic group.

Microbial 16S rRNA compositions were assessed using RNA extracted from the natural chimney structure with or without STPP and the sonication step. The same OTUs were dominantly detected in all 16S rRNA libraries, and neither sonication nor STPP markedly affected subsequent RNA analyses. At the class level, the microbial phylotype composition was significantly dominated by the phylotypes of *Epsilonproteobacteria* (80–98%), followed by the phylotypes of *Methanococci* (0.4–10.1%) and *Deltaproteobacteria* (0.8–6.1%) (Fig. 4). Members of the class *Epsilonproteobacteria* have been dominantly detected in the chimney habitats of most deep-sea hydrothermal fields (12, 31, 32). At the genus level, the phylotypes of *Thioreductor*, *Sulfurimonas*, *Sulfurovum*, and *Lebetimonas* were commonly found as the predominant populations in all three libraries. The most abundantly detected OTU was closely related to the genus *Thioreductor* (30) in all three libraries (Fig. 4). Besides the 16S rRNA sequences of bacteria, sequences closely related to the methanogenic archaea, members of the genus *Methanocaldococcus* (23, 35), were dominantly detected. The relative abundance of *Methanocaldococcus* sequences was significantly decreased by the sonication step (indirect extraction) (Fig. 4), suggesting that the cells of bacteria, particularly *Epsilonproteobacteria*, with a potentially high RNA content were in biofilms and became accessible to TRIzol after sonication.

## Conclusion

There has been a growing interest worldwide in seafloor and subseafloor energy and mineral resources. Deep-sea vent ecosystems including microbial communities are faced with anthropogenic environmental disturbances, and temporal and spatial variabilities in *in situ* microbial diversity and function need to be monitored using a polyphasic approach. Although the STPP amount and sonication step need to be adjusted to each chimney sample, the indirect RNA extraction method developed in this study is simple, rapid, and cost-effective, and may be used for large-scale RNA extraction. This new method may extend analytical methods for microbial communities within deep-sea hydrothermal vent chimneys, and thus may further our understanding of microbial activities in deep-sea hydrothermal fields.

## Supplementary Material
