# Supplementary material for: A Simple and Efficient RNA Extraction Method from Deep-Sea Hydrothermal Vent Chimney Structures
Source: Microbes Environ. 2017 Nov 30;32(4):330–5. doi: 10.1264/jsme2.ME17048 (PMC5745017; doi:10.1264/jsme2.ME17048)
Supplement: Supplementary file 1 [file 32_330_s1.pdf]

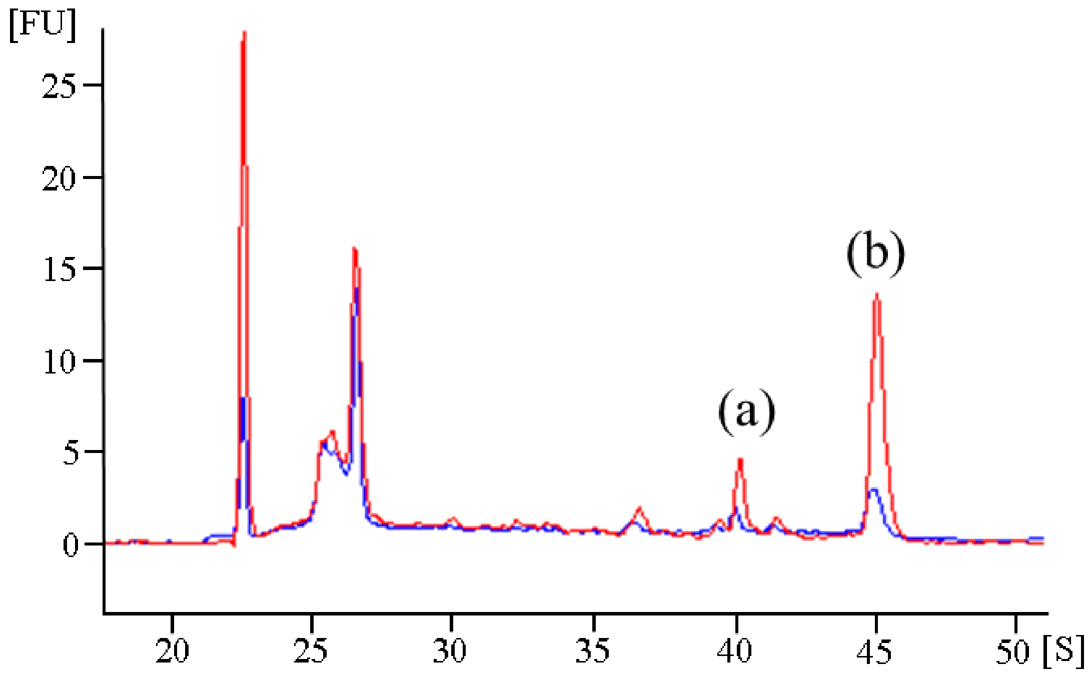

Fig. S1. A Bioanalyzer electrogram. Red and blue lines represent the RNA sample after 0 h and 14 h of mixing with mock chimney, respectively. (a) and (b) indicate 16S and 23S ribosomal RNA peaks.
